# Ending the HIV/AIDS epidemic in low- and middle-income countries by 2030: is it possible?

**DOI:** 10.12688/f1000research.9247.1

**Published:** 2016-09-15

**Authors:** Anthony D. Harries, Amitabh B. Suthar, Kudakwashe C. Takarinda, Hannock Tweya, Nang Thu Thu Kyaw, Katie Tayler-Smith, Rony Zachariah

**Affiliations:** 1 International Union against Tuberculosis and Lung Disease, Paris, France; 2 Department of Clinical Research, Faculty of Infectious and Tropical Diseases, London School of Hygiene & Tropical Medicine, London, UK; 3 South African Centre for Epidemiological Modelling and Analysis (SACEMA), University of Stellenbosch, Stellenbosch, South Africa; 4 AIDS and TB Department, Ministry of Health and Child Care, Harare, Zimbabwe; 5 The Lighthouse Trust, Kamuzu Central Hospital, Lilongwe, Malawi; 6 International Union Against Tuberculosis and Lung Disease, Myanmar Country Office, Mandalay, Myanmar; 7 Médecins sans Frontières, Operational Research Unit (LuxOR), Operational Centre Brussels, Luxembourg, Luxembourg

**Keywords:** HIV, AIDS, UNAIDS, antiretroviral therapy

## Abstract

The international community has committed to ending the epidemics of HIV/AIDS, tuberculosis, malaria, and neglected tropical infections by 2030, and this bold stance deserves universal support. In this paper, we discuss whether this ambitious goal is achievable for HIV/AIDS and what is needed to further accelerate progress. The joint United Nations Program on HIV/AIDS (UNAIDS) 90-90-90 targets and the related strategy are built upon currently available health technologies that can diagnose HIV infection and suppress viral replication in all people with HIV. Nonetheless, there is much work to be done in ensuring equitable access to these HIV services for key populations and those who remain outside the rims of the traditional health services. Identifying a cure and a preventive vaccine would further help accelerate progress in ending the epidemic. Other disease control programmes could learn from the response to the HIV/AIDS epidemic.

## Introduction

On 1 January 2016, we entered the new era of the Sustainable Development Goals (SDGs) that will guide the development agenda of the international community for the next 15 years up to 2030. Within these goals, the international community has committed to a bold agenda (SDG.3.4) of ending the epidemics of HIV/AIDS, tuberculosis, malaria, and neglected tropical infections by 2030 ^1^. This vision deserves widespread support. In this paper, we discuss whether this ambitious goal is achievable for HIV/AIDS by 2030 and what is further needed to accelerate progress towards ending the epidemic.

## Achievements and challenges in HIV/AIDS up to 2015

The Millennium Development Goals (MDGs) were conceived in 2000 with MDG 6 aiming to halt and reverse the spread of HIV/AIDS by 2015 (similar targets were set for tuberculosis and malaria). For HIV/AIDS, this goal was achieved. From 2001 to 2013, the annual incidence of HIV infections decreased by 38%, from 3.4 million in 2001 to 2.1 million in 2013 ^2^. From 2002 to 2013, the annual incidence of HIV infections in children decreased by 58%, with 240,000 new infections in 2013 compared with 580,000 in 2002 ^2^. In some regions of the world, mother-to-child transmission of HIV has been virtually eliminated ^2^. AIDS-related deaths also decreased by 35% between 2005 (when the highest number of deaths was recorded) and 2013 ^2^.

Despite this laudable progress, HIV/AIDS remains a major public health threat. In 2015, at the end of the MDG era, there were still an estimated 2.1 million new HIV infections worldwide, adding up to a total of 36.7 million people living with HIV/AIDS, and there were 1.1 million deaths ^3^. Enormous challenges continue. Although knowledge of HIV status amongst people living with HIV is higher than before, more than half do not know they are infected with HIV ^4^. In many countries, there are key populations in whom HIV incidence and AIDS-related mortality are much higher than in the general population: adolescent girls, sex workers, men who have sex with men (MSM), transgender people, people who inject drugs, prisoners, and migrants ^4^. Structural and legal barriers impede technical progress. For example, same-sex sexual acts are criminalised in 78 countries and are punishable by death in seven ^4^. Sex work is illegal and criminalised in 116 countries and, almost everywhere, illegality and criminalisation envelope persons who inject drugs ^4^. These barriers make it difficult for some of the key populations to access HIV services. Indeed, in some countries in Central Asia, Eastern Europe, the Middle East, and North, West, and Central Africa with limited access to services for key populations, the number of new HIV infections is rising ^4^. In addition, reaching populations affected by conflict and humanitarian crises and migrants (internal and cross-border) is a challenge now and will be in the years to come.

The scale up of antiretroviral therapy (ART) has been one of the major public health success stories of our time, with 17 million people accessing ART by the end of 2015 – 2 million more than the 15 million target set by the United Nations General Assembly in 2011 ^3^. Global coverage of ART reached 46% at the end of 2015, with the greatest gains made in the world’s worst-affected regions of East and Southern Africa. South Africa alone had nearly 3.4 million people on treatment. However, coverage globally is not uniform. Eastern Europe and Central Asia, for example, have seen little expansion in ART scale up in the last few years. Treatment coverage is also often worse among men compared with women, with men frequently initiating treatment late and adhering poorly to medication ^3^.

Finally, for HIV/AIDS, there has been a significant funding gap relative to the resources needed to fully address the epidemic, and the gap is growing. This problem is acute in some low- and middle-income countries where external financing is reducing and domestic resources do not match needs ^2, 4^.

## The response to HIV/AIDS and ending the epidemic

Given the size of the problem and on-going challenges with HIV/AIDS, what makes us think that the goal of ending the AIDS epidemic can be achieved? First, there is compelling evidence that certain biomedical interventions can significantly reduce new HIV infections and AIDS-related mortality. The key measures are the diagnosis of HIV infection and the sustained provision of ART to all those diagnosed with HIV. Treatment for HIV, especially when given immediately after diagnosis irrespective of CD4 count or World Health Organization (WHO) clinical stage, substantially reduces the risk of illness and death in the individual, and this has been shown conclusively in randomised controlled trials ^5, 6^. The benefits of immediate ART also go beyond those for individual patients. Immediate ART reduces the risk of sexual transmission of HIV, this benefit having been demonstrated not only in clinical trials but also in the real world setting ^7, 8^. Immediate ART reduces the risk of acquiring herpes simplex virus type-2 infection ^9^, decreases the risk of tuberculosis ^10^, provides concurrent treatment for hepatitis B infection, and obviates the current problem of poor retention in pre-ART care ^11^. Option B+, the current recommended strategy for preventing mother-to-child transmission, has immediate ART at its core and, by offering lifelong treatment to HIV-infected pregnant and breastfeeding mothers regardless of CD4 cell count or WHO clinical stage, has significant benefits for HIV transmission prevention and maternal health ^12^.

This body of knowledge prompted the joint United Nations Program on HIV/AIDS (UNAIDS) to release new 90-90-90 treatment targets for HIV ^13^. These targets specify that by 2020, 90% of individuals living with HIV will know their HIV status, 90% of people with diagnosed HIV infection will receive sustained ART, and 90% of those on ART will be virally suppressed. If this three-part strategy is achieved, nearly three-quarters of all people living with HIV will be on treatment and virally suppressed by 2020. Modelling studies suggest that this outcome will enable the world to end the AIDS epidemic by 2030, defined as a 90% reduction in both incidence of HIV and AIDS-related mortality ^13^.

### Knowledge of HIV status

The first key measure is knowledge of HIV status. The development of inexpensive, sensitive, specific, rapid, and simple-to-use diagnostic tests has allowed HIV testing to be scaled up, decentralised, and undertaken when necessary by non-laboratory-based personnel. Currently available third-generation rapid tests are able to detect HIV antibodies approximately 21 days after exposure; research is also on-going to reduce false negative tests during this three-week window period ^14^. While the greatest diagnostic yields come from provider-initiated testing and counselling at health facilities, the need to increase case detection at the population level and to diagnose HIV earlier in the course of infection has led to a broad array of community-based approaches targeting asymptomatic people outside of health facilities, including home-based, mobile, workplace, school, self, and campaign-oriented testing ^2, 15^. Self-testing is emerging as a useful and potentially supplementary way to increase the proportion of people who know their HIV status ^16^. Point-of-care HIV testing, using oral fluid rapid tests and carried out by peers, has also been successfully introduced at social venues in Brazil for key hard-to-reach populations such as transgender people, MSM, and commercial sex workers, and this has resulted in improved access to HIV testing ^17^. With all of these methods, though, it is crucial that there is good linkage to ART, and innovative approaches (such as additionally offering home initiation of treatment) need to be pilot tested and scaled up ^16^.

### Safe and effective ART with the response closely monitored

The second key measure is provision of safe and effective ART. Treatment has come a long way since combination therapy was first shown to be effective in reducing morbidity and mortality in patients with advanced HIV infection in developed countries in the mid-1990s ^18^. Costs have plummeted and the current WHO-recommended first-line regimen of tenofovir-lamivudine (emtricitabine)-efavirenz is effective, safe, and non-toxic and is taken as a single pill once a day ^19^. Further work being done on a lowered dose of efavirenz and improved safety of a novel tenofovir prodrug holds promise that this single-dose, one-pill-a-day regimen will become largely devoid of side effects and will require little or no laboratory monitoring in the future ^20, 21^. The development of safe, effective, and non-toxic medication paved the way for the WHO to recommend that ART should be offered to all persons living with HIV regardless of clinical stage or CD4 cell count ^22, 23^. Monthly or less frequent dosing by using long-acting injectable therapeutics (“depo-drugs”) could further improve adherence and treatment outcomes in the future ^24^.

Monitoring the response to ART is crucial. Some countries with limited resources, such as Malawi, have managed through a public health approach to monitor the numbers of people living with HIV who initiate ART and, of those, the numbers who are alive and retained in care, are dead, are lost to follow-up, have discontinued therapy, or have transferred from one ART clinic to another ^25^. While programmatic treatment outcomes are important for assessing effectiveness and for planning the resources needed on the ground, the measurement of viral load is key for assessing whether ART is effective and whether HIV disease is progressing or drug resistance is developing ^2^. Current viral load technologies are laboratory based and costly, so there is an urgent need to invest in, develop, and roll out low-cost, point-of-care viral load tests that can be used to monitor ART at all levels of the health system to determine whether drug adherence is poor (and therefore correctable) or whether a switch in treatment regimen is needed. At the Durban XXI International AIDS Conference in July 2016, it was clear that several new point-of-care viral load tests have already been assessed and should be ready soon to be deployed in the field – this is where investments need to be made with viral load testing replacing CD4 cell count technology.

### Other HIV prevention measures

While scale up and implementation of HIV testing and ART initiation for those HIV infected in different epidemic settings are essential to the global strategy, it is well recognised that a combination response with other well-proven interventions (pre-exposure prophylaxis [PrEP] with antiretroviral drugs, voluntary medical male circumcision, condom promotion, behaviour change programmes, opioid drug substitution, needle and syringe exchange programmes, and outreach programmes for MSM and sex workers) that depend on country and context will be needed to achieve maximum benefit ^2, 26^.

For PrEP, the science is now clear that a co-formulation of oral tenofovir and emtricitabine significantly reduces the risk of HIV infection among non-HIV-infected persons at high risk of acquiring infection ^27^. Randomised controlled trials and open-label extension studies have shown the efficacy of this approach in MSM ^28, 29^, heterosexual men and women in HIV-discordant relationships ^30, 31^, and people who inject drugs ^32^. The studies that have shown no benefit of PrEP have identified poor adherence to medication as the main culprit ^33^. Expanding access to PrEP is therefore not only sound public health policy but also a human rights imperative, and this strong addition to the HIV prevention arsenal has been recognised in the recent WHO Consolidated Guidance on treating and preventing HIV ^22, 23^.

Voluntary male medical circumcision is another proven HIV prevention strategy that was tested in three randomised trials between 2005 and 2007 in South Africa, Kenya, and Uganda ^34– 36^. The results showed that male medical circumcision reduced HIV acquisition in men by about 60%. Other benefits of this surgical approach to HIV prevention include a decrease in incidence of herpes simplex virus type-2 and human papillomavirus infection ^37^. Since 2008, the uptake of voluntary medical male circumcision has increased rapidly, with more than 10 million procedures performed by September 2015 ^26^.

The contribution of different interventions will vary depending on the epidemic setting ^2^. For example, in generalised epidemics, the scale up of ART, especially immediate start of treatment, could contribute the most to a reduction in new infections, followed by condoms, PrEP, voluntary male medical circumcision, outreach to sex workers, and behaviour change communications. In epidemics driven by injecting drug use, the major contributors to a reduction in new infections could come from opioid drug substitution, needle and syringe exchange programmes, and the strategic use of ART. As countries move towards epidemic control targets, innovative and more efficient service delivery models that include improved diagnostic tools and longer-acting antiretroviral drugs will likely further accelerate progress towards ending the epidemic.

### Addressing structural, legal, and social barriers

Several structural factors are recognised as being key to the HIV/AIDS response, and these need to be addressed to maximise the chances of achieving the target goal by 2030 ^2^. For example, the completion of secondary education reduces vulnerability to HIV infection, with the effects particularly seen in girls ^38^. Violence prevention and HIV programming can have potential benefits, especially when integrated into community empowerment schemes that embrace microfinance support. Community mobilisation interventions can help to change behaviour associated with high risk of HIV infection. The many laws, policies, and practices that stigmatise and discriminate against people living with HIV and key populations must be challenged and removed. Civil society and activism, the anchor sheet for years of the HIV/AIDS response, is under threat through restrictive laws and funding cuts, so the global HIV community must show solidarity with civil society and reaffirm its place in the HIV/AIDS response. Young people are those most affected by the HIV epidemic, and countries and initiatives worldwide need to recruit and train the younger generation and use their language and their tools (social media and encounter apps) to leverage the fight against HIV/AIDS. Innovations in science and technology are more likely to be accepted and taken up by the younger generation who now need to take on a leadership role and carry the torch for ending the HIV epidemic by 2030.

## Is there a cure or vaccine to end the AIDS epidemic?

The success of ART has led to the consideration of whether a cure for AIDS might be possible. Although ART causes complete or near-complete inhibition of HIV replication, the virus persists in long-lived infected resting T cells, which contain integrated, transcriptionally latent HIV DNA, and these serve as a reservoir for on-going infection ^39^. Cure in HIV/AIDS is usually defined as *sterilising* (all latent HIV-infected cells are eliminated) or *functional* (latent HIV persists but viraema is very low or absent without the use of ART). The only reported case of a sterilising cure is the Berlin patient, an HIV-infected man given a bone marrow transplant from a naturally HIV-resistant donor for acute myeloid leukaemia ^39^. While an interesting observation, such an invasive intervention could never at present be widely implemented. The focus has therefore been on the possibility of a functional cure.

The first report of a functional cure was that of an infant born to an HIV-infected woman who received ART within 30 hours of birth (the Mississippi baby), with the child having an undetectable viral load after ART was discontinued at the age of 18 months ^40^. This was followed by speculation that very early treatment might prevent the formation of latent reservoirs for HIV, at least in infants with an immature immune system. Unfortunately, recent evidence of viral rebound in the Mississippi child has shattered hopes for this approach ^41^. At present, no cure for HIV is in sight, although the research continues. Ultimately, getting rid of HIV from latent reservoirs will need a combination approach both for activation of the virus and for clearance, a biomedical strategy known as “shock and kill” ^42^.

Some have argued that the only guarantee of a sustained end to the AIDS epidemic will be a combination of non-vaccine prevention methods and the deployment of a safe and effective HIV vaccine ^43^. Ever since HIV was discovered as the cause of AIDS in 1983–1984, intensive work has been undertaken to develop a preventive vaccine. Yet, to date, only three vaccines have completed clinical efficacy trials. The first two candidate vaccines failed their efficacy trials ^44^, while the third vaccine (RV144) showed modest efficacy at 31% in preventing acquisition of HIV infection ^45^. Despite these setbacks and the inherent difficulties in developing an effective HIV vaccine, the science continues with optimism that theory and empiricism will ultimately coalesce for a good end result ^46^.

## Conclusion

We believe that the goal of ending the HIV/AIDS epidemic by 2030 is achievable and should gather momentum. The WHO’s “3 by 5” strategy, aiming to provide ART for 3 million people by 2005, was thought to be wildly over-ambitious and unrealistic by some. However, in reality, it inspired concerted international action and huge progress, leading to momentum that ultimately resulted in 17 million people receiving ART by 2015 ^2^. The current bold ambitious target for HIV/AIDS is similarly needed to inspire, energise, and mobilise communities.

Modelling studies suggest that meeting the 90-90-90 targets using current health technologies will enable the goal of ending HIV/AIDS by 2030 to be achieved, even without a therapeutic means of cure or a preventive vaccine ^13^. We have come to this point because i) the diagnosis of HIV is based on rapid, easy-to-use diagnostic tests that are truly point of care, allowing decentralisation and task sharing and therefore wide coverage, ii) ART is now a single, once-a-day pill that is effective, safe, and relatively non-toxic and hence can be used in asymptomatic persons with or without HIV to both treat HIV and prevent the transmission of infection, and iii) there has been significant engagement by the community affected by HIV resulting in dramatically reduced drug pricing and increased access, significant international donor interest in the form of the GFATM (the Global Fund to Fight AIDS, Tuberculosis, and Malaria) and PEPFAR (the US President’s Emergency Plan for AIDS Relief), and successful programme delivery in many settings.

Diagnosing the millions of people who do not know that they have HIV and starting and retaining those people on ART are daunting prospects and will require increased focus and efficiency including strong political will, scale up of human resources, logistic capacity, better-functioning health systems, innovations in service delivery including community- and patient-driven initiatives, and additional financial resources. Importantly, finding practical ways of reaching those who lie outside the rims of the traditional health services, “the how to deliver”, will need focused attention. This includes populations affected by conflict and humanitarian crises.

Over the past 30 years, funding for the HIV/AIDS response in low- and middle-income countries has risen from almost nothing to $19 billion per annum, although it is estimated that this needs to rise further to $36 billion per annum to achieve the UN goal of ending the AIDS epidemic by 2030 ^2^. In many settings, though, the reality is that less than half of the resources are spent on ensuring access to early treatment and the price tag to optimise treatment coverage may well be within the current funding envelope. It is our opinion that a substantial part of HIV/AIDS funding, including that from the Global Fund and PEPFAR, is spent on non-evidence-based interventions, and this has to change with monitoring and evaluation ensuring that resources are well utilised. Efficient use of funds also means controlling corruption and preventing the deviation of resources to activities other than HIV and health system strengthening.

In summary, in the last decade, the HIV/AIDS community has shown itself capable of rising to a plethora of challenges; in the absence of complacency, we should be able to accelerate progress over the next two decades and end the AIDS epidemic.
